# Chronic Myeloid Leukemia in India

**DOI:** 10.1200/JGO.2015.002667

**Published:** 2016-07-20

**Authors:** Prasanth Ganesan, Lalit Kumar

**Affiliations:** **Prasanth Ganesan**, Cancer Institute (WIA), Chennai; and **Lalit Kumar**, All India Institute of Medical Sciences, New Delhi, India.

## Abstract

**Background:**

In the last decade, the use of imatinib has brought a paradigm shift in the management of chronic myeloid leukemia (CML). In India, imatinib has been available for more than a decade and has been made accessible to all segments of the population because of patient assistance programs and cheaper generic versions. Despite improvements in survival, there are unique challenges in the Indian context.

**Methods:**

We reviewed published data pertaining to CML in India for the period of 1990 to 2016, using PubMed advanced search with the terms chronic myeloid leukemia and India, and included studies that reported on epidemiology, monitoring for therapy, treatment outcomes, and resistance. Additionally, the references in retrieved articles were also reviewed.

**Results:**

Thirty-seven studies were identified. The incidence of CML may be slightly lower in India than in the West, but there was only a single article reporting population-based data. Indian patients presented with more advanced disease. Most centers have access to imatinib as first-line therapy, but there is limited availability of molecular monitoring and second-line therapy. Most of the outcome data were retrospective but seemed comparable with that reported in Western centers. Drug adherence was impaired in at least one third of patients and contributed to poor survival.

**Conclusion:**

Focused prospective studies and cooperative studies might improve the quality of data available. Future studies should focus on adherence, its effects on outcomes, and methods to address this problem.

## INTRODUCTION

Chronic myeloid leukemia (CML) is one of the most common blood cancers. The treatment of CML underwent a dramatic shift with the discovery of imatinib. In fact, CML has served as the poster child for targeted therapy in oncology and remains one of the most successful examples of personalized treatment. In India, patients with CML were traditionally treated with hydroxyurea or busulfan. In the 1990s, the few patients who could afford the cost received interferon-based therapy or stem cell transplantation. When imatinib became available, few patients in India could afford the cost of the innovative product. Subsequently, patient-support programs, such as the Glivec International Patient Assistance Program, and cheaper generics made the medicine almost universally available to patients in the country.

Currently, in most oncology centers in India, newly diagnosed patients with CML begin receiving imatinib. However, the remarkable longevity that these patients have come to enjoy has brought new challenges in their care. Some of these are the need for the sustained availability of drugs; the requirement for strict medication adherence; the ability to recognize and manage long-term physical and psychologic issues, including possible compromises in quality of life; and special issues in pediatric patients. There exists a small but significant proportion of patients who are unable to tolerate imatinib or experience treatment failure. These patients need therapy with more expensive, second-line tyrosine kinase inhibitors (TKIs). In fact, most large centers in the country have patients who experienced treatment failure with imatinib but are now back to receiving older medicines, such as hydroxyurea. Stem cell transplantation, which is the standard therapy for patients with CML presenting with advanced-phase disease, is still out of reach for the majority of Indian patients. In this article, we present the available literature pertaining to CML in India and the unique challenges in its management.

## METHODS

**Fig 1:**
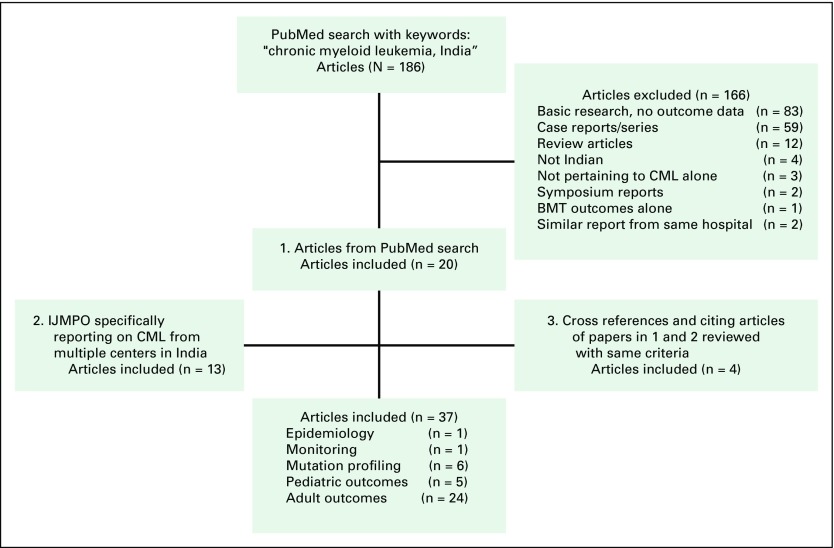
Flow diagram showing the study selection process for the review. BMT, bone marrow transplantation; CML, chronic myeloid leukemia; IJMPO, *Indian Journal of Medical and Paediatric Oncology*.

In this review, we sought evidence pertaining to the following aspects of CML in India: epidemiology, clinical presentation, treatment methods, outcomes of therapy and factors affecting the outcomes, resistance to imatinib in Indian patients, adherence to therapy, and pediatric CML. A literature search was performed in MEDLINE on the PubMed database; articles pertaining to these aspects were chosen and others were excluded. The key words included chronic myeloid leukemia and India, and the search was limited to the English-language literature published from 1990 to 2016 by Indian institutes; 186 articles were selected. Of these, 166 articles were excluded: 83 were basic research or had no correlation to clinical outcomes; 59 were case reports or reports of small series; 12 were review articles or commentaries; four did not report on Indian studies; three did not pertain to CML; two were symposium reports; one pertained to bone marrow transplantation outcomes; and two were excluded because the outcomes from the same set of patients were reported in a subsequent publication, which was included in the analysis. Twenty studies were included for further analysis based on this search.^1-20^ A special issue of the *Indian Journal of Medical and Pediatric Oncology* reported on the outcomes of CML in Indian centers; all these articles were separately reviewed using the same criteria, and an additional 13 papers were included.^21-33^ The references of all publications were reviewed, and an additional four studies were identified and included.^34-37^ Studies in which there were no data on patients who died or analysis was confined to patients who were followed-up in the outpatient department were also excluded.^38^ Of the 37 studies thus reviewed, one was related to epidemiology,^36^ one concerned efforts to set up monitoring facilities in India,^7^ six reported on the mutation profile in Indian patients receiving imatinib, five reported outcomes in pediatric patients, and 24 discussed outcomes in adult patients (Fig 1).

On the basis of data from these studies, the following aspects were reviewed: epidemiology, clinical features, treatment and outcomes, and data on resistance to TKIs and adherence to therapy. The relevant data are presented in the following sections, and the published studies are compared with other international studies. A brief update on the availability of imatinib and other TKIs is provided to give perspective and context to the review.

### Epidemiology of CML in India

The annual incidence of CML in India was originally reported to be 0.8 to 2.2 per 100,000 population.^39^ However, these were estimates and may not represent the true incidence because most of the data from population-based registries in India report myeloid leukemia as a single entity without separating the acute and chronic forms.^40^ A recent study from the Mumbai Cancer Registry specifically examined CML and reported an age-adjusted rate (AAR; per 100,000) of 0.71 in males and 0.53 in females. The incidence varied across age groups, with an increased incidence in older individuals. Interestingly, there was a decreasing trend in the incidence of CML over the years.^36^ The incidence reported in this study was lower than that reported in the United States (AAR, 1.75) and Australia (AAR, 1.2) but is close to that reported in many centers in Europe.^41-44^ Other Asian countries have also reported a lower incidence of CML compared with the United States, and the reasons for these variations are unclear (Table 1).^39,45^

**Table 1:**
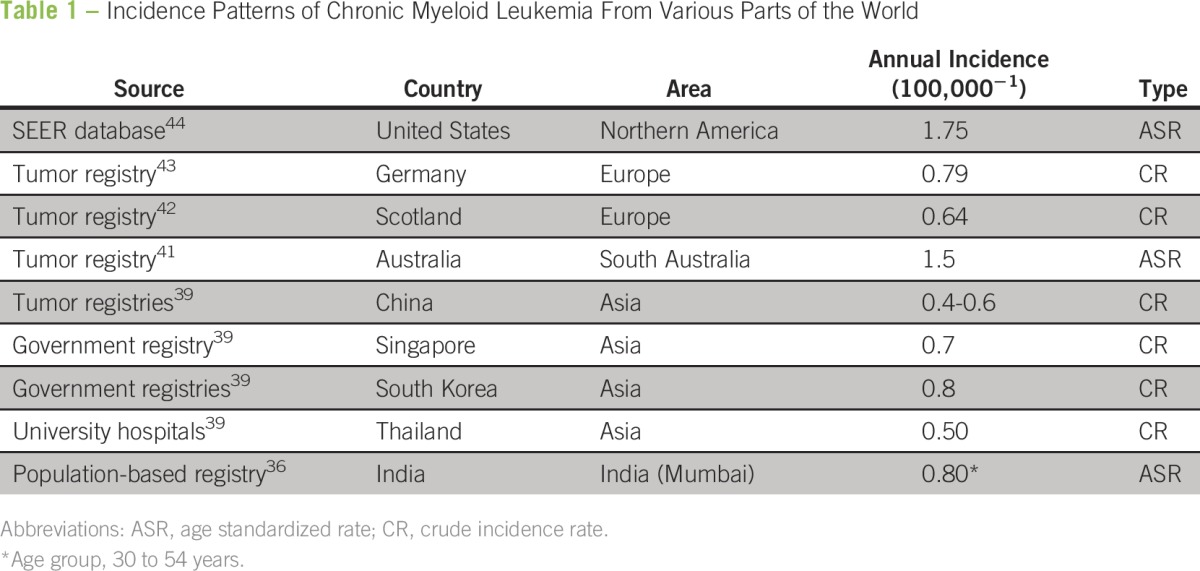
Incidence Patterns of Chronic Myeloid Leukemia From Various Parts of the World

### The TKI Story in India

Imatinib was made available to most patients with CML in India through a Novartis-sponsored program that was administered by the MAX foundation, called the Glivec International Patient Assistance Program.^46^ This program continues to remain active in many major centers in the country. In addition, the availability of generic imatinib has also contributed to cheaper access to the drug. Retrospective analyses have shown that equivalent response rates can be achieved with generic imatinib.^22,29^

Dasatinib and nilotinib are the second-line TKIs currently available in India. Although data from randomized trials suggest that first-line use of second-line TKIs can lead to faster and deeper molecular responses, the higher cost limits their use in newly diagnosed patients. There are few reports detailing the use of second-line TKIs in India.^6^

### Monitoring of Therapy in CML

The cost of monitoring by conventional cytogenetics, fluorescent in situ hybridization, and polymerase chain reaction (PCR) are Indian rupee (INR) 500 to 1,000, INR 2,000 to 3,000, and INR 5,000 to 7,000, respectively (Table 2). Conventional cytogenetics requires frequent bone marrow punctures, which are not acceptable to patients. An alternative is fluorescent in situ hybridization for the *BCR-ABL* translocation, which can be performed from the peripheral blood and is more sensitive than karyotyping.^47^ Reverse transcriptase-PCR is currently the most sensitive technique available, and the results correlate well with long-term outcomes. However, PCR techniques are plagued with interlaboratory variability, which limits interpretation and comparison of data. To counter this, international standardization has been proposed, but there are few laboratories in India that have standardized testing available.^7^

**Table 2:**
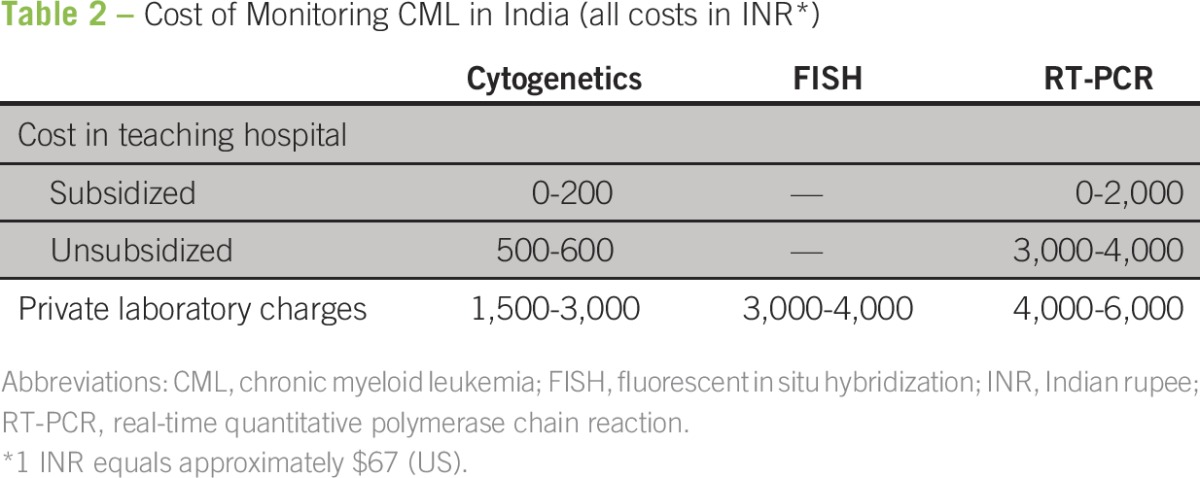
Cost of Monitoring CML in India (all costs in INR*)

The practical relevance of frequent testing, which is expensive in itself, in the context of limited access to second-line options can be debated in the Indian context. It can be argued that little is achieved by molecular testing in a poor patient who otherwise has complete hematologic response (CHR)/complete cytogenetic response (CCR) with imatinib therapy because there is no option of switching to another TKI if there is poor molecular response.^8,48^ However, even in these patients, an increased dose of imatinib might help at least a quarter of patients to achieve better responses. This might be considered an argument for performing serial monitoring, even in resource-limited settings. Efforts are being made to make cheaper technology available for molecular monitoring of CML.^48^

### Clinical Presentation

Patients with CML in India have key differences in the presenting features compared with Western patients (Table 3). In India, it is unusual to diagnose CML in an asymptomatic patient, and a higher proportion of patients have organomegaly, constitutional symptoms, and high-risk Sokal scores.^27,38^ Although lower socioeconomic status has been correlated with a higher-risk Sokal score at presentation, there is only a single study examining this issue.^27^ Other analyses from India question the relevance of the Sokal score itself in our population.^8,19,22^

**Table 3:**
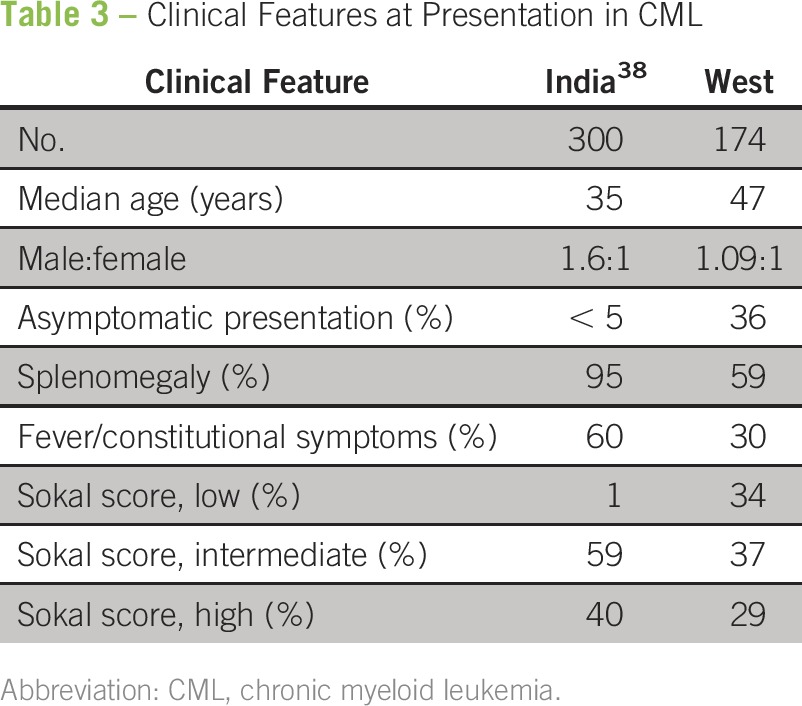
Clinical Features at Presentation in CML

Although multiple articles using hospital-based data have suggested that there may be a younger age of presentation in CML in India, population-based studies have refuted these claims.^36^ The study from the Mumbai Cancer Registry showed an age distribution similar to that seen in the West, with the highest incidence in the age group of 55 to 74 years.^36^

### Treatment and Outcomes of CML in India

#### Chronic phase.

Before the availability of imatinib, the outcomes of CML reported in various centers in India were dismal. Most centers used hydroxyurea for therapy, considering the limited availability of stem cell transplantation facilities and the high cost of interferon.^22,38,40^ The availability of imatinib and support programs has created a sea change in the treatment of CML in India. Responses to imatinib in Indian patients seem similar to those reported in the West, at least in patients who can take the medicine consistently (Table 4). Reports from centers treating patients in the higher socioeconomic strata, where logistical constraints are less likely to affect adherence behavior, have shown outcomes similar to those reported in Western populations.^24^ Generally, CCR rates of 60% to 80% have been consistently reported from various centers in the country.^8^ There are limited published data on the use of second-line TKIs.^6^

**Table 4:**
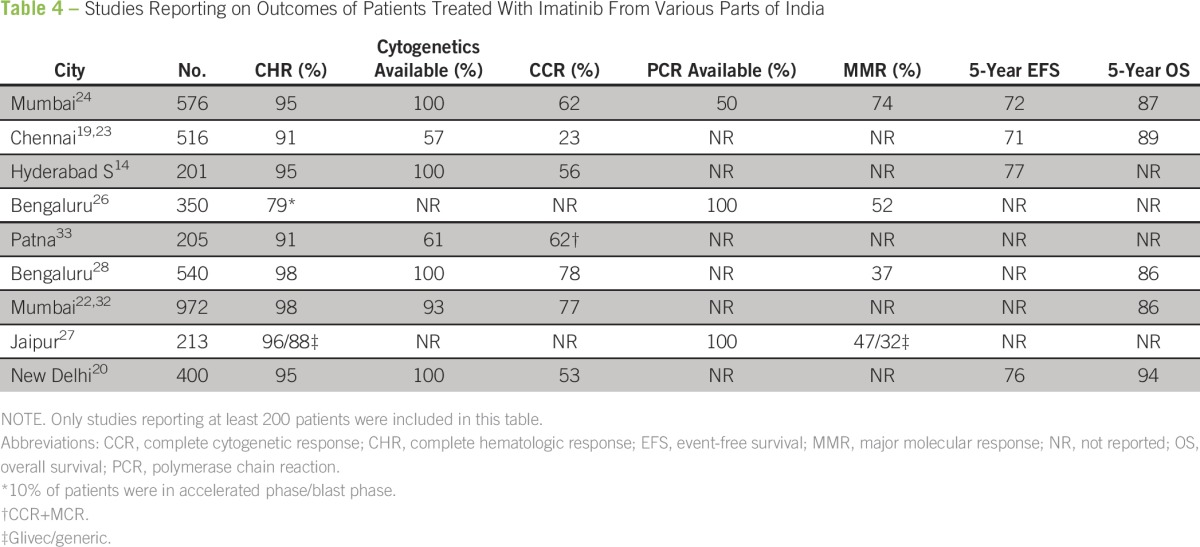
Studies Reporting on Outcomes of Patients Treated With Imatinib From Various Parts of India

#### Accelerated phase and blast phase.

Imatinib can be used to treat CML-accelerated phase, but the outcomes are worse.^49,50^ One of the few reports of advanced-phase CML in India showed a median survival of 61 months for CML-accelerated phase and a survival of 14 months for CML-blast phase.^30^ Second-line TKIs used upfront may improve outcomes, but, again, the access is limited. Stem cell transplantation offers the best chance for a long-term cure in a patient with CML-blast phase as soon as a reversion to chronic phase is achieved (which happens in 30% to 50% of patients treated with TKIs).^49^

#### Management of imatinib treatment failure.

The best approach to imatinib treatment failure is to change to a second-line TKI when the patient can afford the medicine. The other option is to increase the dose of imatinib, which may still improve the response in those patients who have achieved suboptimal outcomes. CCR can be attained in 50% of patients who do not achieve CCR or lose CCR, but for those with loss of CHR or nonachievement of CHR, the chance of attaining CCR was only 18%.^16,51^ One study reported that approximately 25% of patients who experienced treatment failure with imatinib can regain optimum cytogenetic responses with dose escalation.^23^

#### Adverse events after treatment with TKIs.

The profile of adverse events in India mirrors that in Western published literature. Certain unique adverse events have been described in the Indian population, such as extensive hypopigmentation of the skin.^19,35,52^

### Resistance to Imatinib

Although multiple reasons may be identified for imatinib resistance, mutations in the *BCR-ABL* gene is a common problem.^53^ Studies from India have identified mutations in 45% to 50% of patients showing resistance to imatinib.^3,12^ These mutations may be higher in patients with more advanced disease.^3^ In a large report involving data from more than 1,000 patients from various parts of India, mutations were identified in 41% of the samples, which were analyzed by a private laboratory.^25^ This study showed that nine common mutations, which accounted for 85% of the total mutations seen in the kinase domain of the *BCR-ABL*, occurred in a different frequency than those reported in the West.^25^ A separate, single-institution study has shown similar findings.^16,51^

### Patient Follow-Up and Adherence

An important component of CML therapy is the requirement of adherence to medications.^54^ Although most patients tolerate imatinib fairly well and serious adverse events are rare, even after prolonged intake, there are many minor adverse events of imatinib that, over prolonged periods, can significantly compromise the quality of life and might even promote nonadherence to therapy.^54-56^ Nonadherence to therapy occurs in up to one third of patients with CML treated with imatinib.^19,57-60^ Nonadherence, even to a minor extent, can prevent optimal outcomes.^19,22^ Because imatinib is the only option for treatment in the majority of Indian patients, it follows that efforts must be made to make maximum and optimum use of this medicine in a given patient. Hence, time must be spent during clinic follow-up visits to formally address this topic with patients and to reinforce the importance of being compliant with treatment. Studies have clearly shown that simple education and reinforcement can greatly enhance medication adherence.^61^

### CML in Indian Children

Worldwide, approximately 3% of childhood leukemias are CML, with a 10% incidence in children ages 5 to 20 years.^62^ The majority of childhood CML in India occurs in patients older than 12 years of age.^35,38^ In the pre-imatinib era, a dismal median survival of approximately 13 months was noted.^38^ Currently, survival outcomes are similar to those reported in the West.^35^ Most of the reports of pediatric CML in India pertain to patients with no access to second-line TKIs or allogeneic transplants and, as such, reflect the natural history of pediatric CML treated with imatinib alone.^35,37^ Children can have growth retardation due to long-term imatinib therapy, which has also been reported in Indian studies.^9^

In conclusion, we attempted to collect all the relevant literature concerning CML in Indian patients, particularly with respect to treatment outcomes. However, almost all the studies reviewed presented a retrospective data analysis. Studies also covered multiple treatment eras and lacked molecular and cytogenetic monitoring details. Given these limitations, we attempted to summarize the salient findings of the larger studies and have tried to provide a consolidated picture of the status in India.

CML in India has certain unique features. The overall incidence may be slightly lower than that reported in the West, but data are not available for most populations; hence, definite conclusions are not possible. Most of the Indian patients with CML present with symptoms, possibly with higher-risk disease. Most centers in the country currently use imatinib as first-line therapy. The drug is available at low cost or free of charge at most large centers. Patients receiving long-term imatinib therapy face major challenges, especially with regard to drug adherence. There is evidence that poor adherence impairs the outcomes in these patients. In addition, in the Indian context, where there is limited access to second-line TKIs, optimizing drug adherence could help prevent a significant number of treatment failures and improve survival. Future studies and efforts should address these concerns.
